# The Gender Gaps in Time-Use Within Italian Households During 2002–2014

**DOI:** 10.1007/s40797-022-00211-5

**Published:** 2022-10-21

**Authors:** Francesca Barigozzi, Cesare Di Timoteo, Chiara Monfardini

**Affiliations:** 1 University of Bologna, CHILD-CCA and IZA, Bologna, Italy; 2 Glovo, Barcellona, Spain

**Keywords:** time use, gender gaps, childcare, household work, D13, J13, J22

## Abstract

How are Italian parents sharing family duties when they are both working full-time? We estimate gender gaps in the allocation of time by young Italian couples with children and document their trends over the years 2002–2014, disentangling time use on weekdays and weekend days. We show that the gaps in time devoted to *Market work* and *Household work* have narrowed over the years 2002–2014, while the gap in *Basic childcare* and *Leisure* remained almost constant. But the negative gap (females-males) in *Market work* shrunk much more than the positive gap in *Household work* (46% against 25%) The best-case scenario for gender parity is the one in which partners share similar work responsibilities and are relatively young; however, we show that family duties remain heavily unbalanced across gender in this sample. In 2014, full-time working mothers devote to *Total work* (paid and unpaid) 11 h per week more and to *Leisure* 9.7 h per week less than their partners. On the positive side, the gender gap in *Quality childcare* exhibits a reversed sign, which is driven by fathers’ engagement on weekend days.

**Supplementary Information:**

The online version contains supplementary material available at 10.1007/s40797-022-00211-5.

## Introduction

Gender imbalances in family work and informal childcare are at the heart of the current economic debate. It is widely recognized that the increase in women’s participation in the labour force that occurred over the past decades in developed countries has not translated into a proportional increase in men’s presence in household activities. In their recent book “Gender and Time Use in a Global Context,” Connelly and Kongar (2017) state that “despite the cliché, time really is the ultimate scarce resource and how we use our time defines who we are and what we produce.” Time use surveys based on time diaries collect detailed data on the whole set of activities performed by the respondent during the 24 h of the reference day and are increasingly recognized as a fundamental tool for understanding time allocation decisions, providing a more accurate and reliable measure with respect to that obtained through retrospective questionnaires; see Sevilla (2014). A considerable advantage brought in by time use data is that they allow observing time allocation outside the labor market (in household chores, child and adult care, and leisure), a possibility which -according to Hamermesh and Pfan (2005)- opens up new perspectives for the economic analysis of household behavior seminally introduced by Becker (1965).

There is indeed ample evidence from various European Countries and the US pointing to considerable gender differences in time use and documenting that women are poorer than men in terms of time devoted to leisure (see, among others, Bittman and Folbre 2004; Craig and Mullan 2011; Anxo et al. 2011; Gimenez-Nadal and Sevilla 2012; Burda et al., 2013; Kolpashnikova et al.; 2018). Gálvez-Muñoz et al. (2011) offer a cross-national comparison of European countries based on the Harmonised European Time-Use Survey (HETUS) data referred to years from 1998 to 2003 and argue that unpaid care work is at the core of gender inequality in all countries. They show that, on average, women work in paid plus unpaid activities longer each day than men; and that countries with the largest discrepancy of at least 1 h of work per day between men and women are the Eastern European and the Mediterranean countries. Gimenez-Nadal and Molina (2020), using 2010 HETUS and Multinational Time use (MTUS) data, document negative (female-males) gaps in market work and positive gaps in unpaid work in all countries, and that these gaps are lowest in Nordic countries and largest in Mediterranean ones.

Italy has gathered interest in relation to this specific topic, standing out as a negative benchmark in official statistics^1^ and in comparative studies. Anxo et al. (2011) compare gender gaps in time use during the life cycle in France, Italy, Sweden, and the US. Analysing the 2002–2003 Italian Time Use Survey, they find that Italy presents the largest gender gap in time use along all stages of the life course. As an explanation for the difference between the four countries, the authors point to Italian strong social norms on gender roles.

Indeed, in Italy, gender roles are still shaped in a traditional way, especially when the children are very young. The World Value Survey depicts Italy as a country where traditional values about gender roles are still very strong (see http://www.worldvaluessurvey.org/wvs.jsp). 51% of the Italian population fully agrees with the claim that “The most important role of a woman is to take care of her home and family,” and, Italy ranks 15 out of the 29 European countries ordered according to conservative views about gender roles (see the 2018 Report on Equality Between Women and Men in the EU).

In addition, in Italy, public expenditure on early childhood and educational care is only 0.6% of country-specific GDP, against an average of 0.8% in OECD countries; see Olivetti and Petrongolo (2017). This translates into inadequate availability of public childcare services: available vacancies in (public and private) daycare centers only cover 27% of Italian children; see ISTAT (2021). To sum up, both rationed childcare services and a strong social norm about the mother as the main caregiver and responsible for household chores contribute to Italian mothers’ heavy burden on family duties.

In this paper, we provide up-to-date estimates of gender differences in the allocation of time in Italy using data from three ISTAT “Use of Time” surveys: 2002-3, 2008-9, and 2013-14; the latter being the most recent survey available. We look at time management across different time categories for the full 24-hour-spectrum, while documenting the evolution of intrahousehold time allocation differences across thirteen years. Consistently with recent related research (Aguiar and Hurst 2007; Gimenez-Nadal and Sevilla 2012), we focus on time allocated to the following categories: *Market work, Household work*, *Childcare*, and *Leisure*. We run descriptive OLS regression models and estimate time gender gaps and their evolution, both unconditional and conditional to a set of individual observed characteristics available in the survey.

In our study, we focus on the following four dimensions.

First, we study the sample of full-time working parents with young children, in which individuals are likely to be characterized by less traditional gender attitudes. To see why consider that both partners have a full-time job, hence they are both contributing to the family income in a relatively balanced way, and we expect the bargaining power of the two partners in the couple to be relatively close to equality. In addition, in our sample, individuals are relatively younger and more educated than the average Italian person, which is likely to imply that partners have been exposed to a more diverse and less traditional cultural environment and are therefore more mindful of gender parity. However, our analysis shows that gender disparities remain huge.

Second, we analyze time devoted to *Childcare*, disaggregating it into *Basic childcare* and *Quality childcare* time, following the definition of the latter provided in Price (2008) and Boca et al. (2017). As we explain in detail in Sect. 3, *Quality* time with children refers to time devoted to all activities that contribute to their education and mental growth, like helping them with homework. Instead, *Basic childcare* refers to time devoted to all activities carried out for children’s basic needs, like feeding them. The distinction is important when analysing gender gaps in time use because basic care is traditionally perceived as a women’s activity and is less psychologically rewarding for fathers. We expect that a fall in the gender gap in *Childcare* will first show up in *Quality* time and then will possibly be followed by a reduction in the gap in *Basic childcare*.

Third, following Bloemen et al. (2010) and Henz (2019), we look separately at weekdays and weekend days. During weekends individuals can allocate their time in a more flexible way, hence changes in time allocation over time due to evolving attitudes and gender norms (with increased attention to gender equity) are likely to appear first on weekend days than on weekdays. Specifically, housework and childcare have a lower opportunity cost during weekend days, and we expect fathers to be more active in family duties on weekend days than on working days. In line with our intuitions, we document an overall larger engagement of fathers during weekend days, but this change affects the gender gap substantially only in the case of the specific time category *Quality childcare*.

Finally, we estimate heterogeneous gender gaps by education level (individuals with and without a university degree) and age ranges (25–34; 35–44; 45–54), thus capturing interesting patterns in behaviors.

Our work relates to studies providing gendered analyses of time use in Italy and other countries. Among others: Bloemen et al. (2010); Craig and Mullan (2011); Garcia-Roman and Cortina (2016); Evrim (2016); Zannella et al. (2019a) and (2019b). Some of these studies adopt a comparative approach; others provide trend implications, many of them focus on individuals living in couples, and very few use the weekend/weekday distinction and disaggregate *Childcare* into *Basic* and *Quality* time. A couple of studies incorporating the last Italian time use survey were recently developed in parallel with our research: Zannella and De Rose (2019a) and (2019b). We discuss those and other papers in the following Section devoted to the related literature.

Our main findings about trends in time allocation gaps across genders in Italian families with full-time working parents are the following. Over the thirteen years under study, gender gaps (females-males) in *Market work* and *Household work* narrowed, while the gender gap in *Basic childcare* and *Leisure* remained basically constant. However, the negative gap in *Market work* shrunk much more than the positive gap in *Household work*: 46% against 25%. The latter is the result of changes in the behaviors of both members of the couple. Specifically, men decreased the time devoted to *Market work* and slightly increased the time devoted to *Household work*. On the other hand, women decreased the time devoted to *Household work*. As for *Market work*, women’s trend crucially depends on their level of education. Specifically, mothers without a university degree increased their labor supply, contrary to mothers with a university degree who decreased their *Market work*.^2^ As a result, in 2014, a gap of more than 10 h per week exists between the time devoted to this time category by the two groups of women. Both men and women increased the time devoted to *Basic childcare* and the time devoted to *Quality childcare*. Men increased particularly the time allocated to *Quality childcare* so that in 2014 the sign of the gender gap in this category turned from positive to negative.

Considering the total workload obtained summing *Market work*, *Household work*, and *Basic* and *Quality Childcare*, the estimated weekly figure is 60 h for a full-time working woman (25 h of *Market work* plus 35 h of *Household work*, *Basic and Quality childcare*) against the 49 h of her partner in 2014. Simply put, the increased involvement of women in the Italian labor market, driven by mothers without a university degree, went hand in hand with an increase in their informal childcare provision and has not been followed by a parallel growth in the participation of their partners in home duties.

Overall, we show that the sharing of family duties is dramatically uneven, despite the relatively young age of individuals belonging to our sample and, more importantly, despite the similar work responsibilities characterizing the two partners. In 2014, full-time working mothers devote to *Market work* 19% less time than their partners (-1.6 h per day) during weekdays, which documents that the gender gap in *Market work* is closing for full-time workers (the drop of the gap with respect to 2002 is about 55%).^3^ However, always on weekdays, women still devote 264% more time than men (+ 2 h per day) to *Household work*, 125% more time (+ 0.6 h per day) to *Basic childcare*, and 33% more time than their partners (+ 0.13 h per day) to *Quality childcare.* This translates into almost − 1 h per day less *Leisure* for full-time working mothers. The situation improves during weekends when male partners contribute more than they do during weekdays to both *Childcare* and *Household work.* Interestingly, partners of full-time working mothers are the main providers of *Quality childcare* during weekends, meaning that the gender gap is reversed for this time category. Despite this, during weekends, full-time working mothers still devote 140% more time to *Household work* (+ 2 h per day) and experience 29% less time of *Leisure* (-1.6 h per day) than their partners. The gap reversal for *Quality care* during weekends is a positive signal about fathers’ involvement in kids’ education, and it could indicate that the gender gap in *Quality childcare* is likely to disappear in a near future also on weekdays. However, the large and persisting gender gap related to home duties is alarming and documents the strength of traditional gender roles in the household in Italy.

The rest of the paper is organized as follows. Section 2 illustrates the related literature; Sect. 3 describes our dataset and provides the definitions of the variables; Sect. 4 presents the empirical strategy and the OLS regression results on the selected categories of activities for full-time working parents, disaggregated by weekdays /weekend days. Section 5 concludes.

## Related Literature

This section is organized into four parts. We present evidence on (1) international trends in time use; (2) gender gaps in time use for individuals living in couples; (3) papers that use Italian time-use data; (4) time use during the Covid pandemic.

### International Trends in time use

Aguiar and Hurst (2007) document trends in the allocation of time within the United States and find a dramatic increase in *Leisure* time for men and women. Specifically, leisure for men increased by roughly six to nine hours per week, driven by a decline in *Market work* hours, and for women by roughly four to eight hours per week, driven by a decline in *Household work* hours. More recently, Craig and Mullan (2013) conducted a gender comparison of parents’ leisure time in Australia, the United States, France, Italy, and Denmark. They conclude that in all countries, both the quantity and quality of leisure time favor fathers.

Fisher et al. (2007) document that total work is declining for men and women in the same way in the US, so the gap in domestic work persists. Similarly, Kolpashnikova (2018) unveils a persistent traditional gender gap in housework in the US. Gimenez-Nadal and Sevilla (2012) compare trends in time allocation in Australia, Canada, Finland, France, the Netherlands, Norway, and the UK. They document general decreases in men’s market work coupled with increases in men’s unpaid work and childcare, as well as increases in women’s paid work and childcare coupled with decreases in unpaid work. Fang and McDaniel (2017) show that *Household work* per person has declined in both the US and European countries over the past 50 years and that female time allocation contributes more to the difference in time allocation per person between the US and European countries than male time allocation does. Gimenez-Nadal and Sevilla (2014) show that, in Spain, female market work increased and that female nonmarket work decreased.

Our evidence from Italy is only partially in line with the above picture and dramatically depends on parents’ level of education. Full-time working fathers substantially decreased *Market work* and increased *Household work* and *Childcare*, but only fathers with a university degree also increased their *Leisure* time. On the other end, full-time working mothers decreased *Household work* and increased time devoted to *Leisure*, but we observe an increase in *Market work* only in the case of mothers without a university degree. Interestingly, low-educated full-time working mothers in 2014 devoted to *Market work* 10 h per week more than their highly-educated counterparts. In addition, we observe that the gender gap in *Leisure* significantly decreased for low-educated parents, while the gap increased for parents with a university degree.

### Gender gaps in time use for Individuals Living in Couples

Sevilla et al. (2010) find that a Spanish woman’s relative share of housework fails to decrease with her relative earnings beyond the point where her earnings are the same as her husband’s. In contrast, a woman’s share of childcare time displays a flat pattern over the distribution of her spouse’s relative earnings. Álvarez and Miles-Touya (2019) focus on the share of household duties in Spanish dual-earner couples and show that a husband’s non-working day leads to an almost equal distribution of housework, whereas a wife’s non-working day leads the wife to perform most of the household tasks.

Guryan et al. (2008) and Gimenez-Nadal and Molina (2013) document a positive educational gradient in time devoted to childcare activities in the U.S., and in Spain and the UK, respectively. Evrim (2016) shows that the gap between high- and low-educated U.S. parents’ investment in quality time with children has widened, and fathers are less and less engaged with their children in households with low-educated mothers.

Craig and Mullan (2011) conduct a cross-national study of mothers’ and fathers’ relative time in childcare in Australia, Denmark, France, and Italy. They show that the average total parental childcare time is the longest in Australia, followed by Denmark, Italy, and then France. The same ranking holds for the time devoted to childcare by fathers, while mothers in Denmark, Italy, and France provide a similar amount of care. In all four countries, mothers spend more time performing childcare than their partners, with fathers spending only 35% (Denmark) and 25% (France) of their spouses’ care time. Garcia-Roman and Cortina (2016) show that Spanish mothers spend more time with children than fathers do and that the employment-status variables are the most determining factors. Couples that share similar jobs and education levels have lower differences in the time that fathers and mothers spend with their children. However, the differences remain high, and mothers are still the main caregivers in all types of households. Henz (2019) documents the stalling of the transformation of the father role and progress towards gender equality in the home in the UK. In addition, father involvement on weekend days continues to diverge between high and low-status groups.

In line with evidence from other countries, our paper documents that the Italian gender gap in *Household work* decreased in past years, especially among low-educated parents. As for *Childcare*, we show that Italian parents increased the time devoted to this time category and that highly-educated parents, especially mothers, provide more childcare than low-educated parents. Disaggregated data show that, while Italian mothers provide more (basic and quality) childcare than their partners during the days of the week, the involvement in quality childcare by Italian fathers increased and, in 2014, the gender gap in this time category changed its sign in weekend days.

### Italian time-use data

Bloemen et al. (2010) analyzed the time simultaneously allocated by Italian husbands and wives to *Market work*, *Childcare*, and *Housework* in 2002–2003. They find that men married to more highly educated women spend more time with their children, but patterns differ substantially between weekends and weekdays. In addition, their analysis suggests that the time devoted to childcare by the two parents is complementary. Carriero and Todesco (2018) investigated whether Italian women’s ability to assert their egalitarian beliefs is linked to having sufficient personal resources in economic and cultural terms in 2013–2014. They find that the effect of her gender ideology is strongest when a woman earns roughly as much or more than her partner and when she holds a college degree. When the woman’s income is lower than the man’s, the effect of women’s gender ideology is quite small. If she does not have a degree, her egalitarian attitude will not translate into less housework.

Zannella and De Rose (2019b) focus on the impact of the economic recession on the time allocation of Italian females and males using the same three surveys we use. Zannella and De Rose (2019a) are more related to our paper. They use the 2013–2014 survey and focus on time transfers in the couple. They show that women continue to be net donors of time within the family and perform the bulk of total work (paid and unpaid) within the couple. Households where both partners are unemployed or where only the woman has a market job show the highest levels of inequality, with women contributing to about 70% of the couples’ total working time.

Overall, our analysis confirms results from previous literature using the same data. Moreover, by disaggregating data in time use on weekend days/days of the week, time use by highly-educated/low-educated parents, and time use by individuals belonging to different ages classes (25–34; 35–44 and 45–54), we also provide additional evidence.

### Trends in time-use Gender Gaps and the Covid Pandemic

Biroli et al. (2021) document a great increase in the proportion of shared childcare and household work in Italy, the UK, and the US. However, mothers still devote more time to household work than their partners, while fathers specialize in grocery shopping. In addition, mothers took care of the incremented homework supervision due to school lockdown while fathers took the responsibility for children’s play - both activities being classified as quality childcare in our paper. Del Boca et al. (2020) studied Italian couples in which both partners were working before the pandemic, 67% with children below 14 years of age. They show that men reduced their housework both when working at the usual place and when working from home compared to when not working. The same is not true for women who did not reduce housework when working from home. Childcare and homeschooling are more equally shared within the couple than housework activities. The authors replicated the study in 2021 and found that results on gender differences in the allocation of housework, childcare, and homeschooling are consistent in the first and second waves of the pandemic; see Del Boca et al. (2022).

Brini et al. (2021) document a reduction in dual-worker couples, an increase in not-working couples, especially among the lower educated, and an increase in female breadwinning. Bettin et al. (2022) conclude that, in Italy, the lockdown did not produce the “she-cession” experienced in other countries. Corsi and Ilkkaracan (2022) provide a survey of the ongoing research on the effects of the COVID-19 pandemic on gender gaps in paid and unpaid work. Profeta (2020) focuses on policies supporting gender equality in times of COVID-19 and explores whether women’s leadership promotes successful measures.

Finally, Mangiavacchi et al. (2021) use data from a real-time survey collected in April 2020; their sample is composed of working parents, with the woman in the age range 25–64. They show that the lockdown had a balancing effect on the parents’ division of household tasks, reducing the mothers’ share of housework and childcare. In addition, time devoted by Italian fathers to homeschooling, and other quality childcare activities increased substantially during the lockdown.

Overall, results from studies on the effects of the COVID-19 pandemic on gender gaps in time use in Italy appear to be consistent with the decreasing trend we documented for gaps in time devoted to *Market work*, *Household work*, and *Childcare*. In other words, this early evidence suggests that the pandemic did not arrest the process of a narrowing gender gap in time use.

## Data and time Categories Definition

We use the “Indagine Multiscopo sulle famiglie - Uso del tempo” developed by ISTAT and pool the *2002–2003*, the *2008–2009* survey and the *2013–2014* survey. For simplicity, in the rest of the paper, we refer to the three surveys as 2002, 2008, 2014. After a careful analysis, we decided to disregard the previous 1988-89 survey since it adopts a classification of the time activities that is only partially consistent with the one of the subsequent surveys, making the comparison across time arduous. In the Appendix, we report the main characteristics of the three surveys we use.

The information gathered in these surveys was collected through direct interviews and through the compilation of a diary where individuals were asked to list all the activities performed during the day and their duration. Within the diary, each respondent had to describe, using her/his own words, the various activities conducted every 10 min with the possibility to highlight a primary and secondary activity.

Following Aguiar and Hurst (2007) and Gimenez-Nadal and Sevilla (2012), we divided time use in the whole 24 h spectrum into seven different macro-categories, splitting some of them into more specific sub-categories, as follows:

1. *Market work*: it includes all time spent at working in the paid sector or main job, second jobs, and overtime. It also includes breaks.
2. *Unpaid work*: all the activities listed in this category might be performed by a third person through a salary or a paid service. It includes two sub-categories:
  2.1. *Household work*: any time spent on meal preparation and clean-up, doing laundry, ironing, dusting, vacuuming, indoor household cleaning, indoor design, indoor maintenance and elderly-care;
  2.2. *Purchase of Goods*: any time spent in obtaining goods and services like grocery shopping.
3. *Childcare*: it includes all the time devoted to child-care. We distinguish between:
  3.1. *Basic childcare* which includes activities like feeding and food preparation, washing, changing children, putting babies to bed or getting them up, babysitting, medical care for babies and so on.
  3.2. *Quality childcare* which is instead related to children education and mental growth. It includes activities like helping with homework, reading books to children, playing games with them and so on.
4. *Self-care*: it lists all the activities related to personal physical needs and basic necessities.
  4.1 *Sleeping*: includes sleeping. 
  4.2 *Other self-care*: it includes all the other self-care activities, like eating, dressing and so on. 
5. *Voluntary work*: it includes religious and voluntary activities.
6. *Leisure*: It includes all time spent on entertainment, social activities, relaxing and recreational activities which are pursued solely for the direct enjoyment such as watching television, sports, socializing, visiting museums, general out-of-home leisure and the like.
7. *Study*: includes study activities.

Following previous literature, we focus on the following activities when they are carried out as primary activities:^4^*Market work, Household work*,^5^*Basic childcare, Quality childcare* and *Leisure*.

Table 1 describes the detailed activities we included in each category, as coded by ISTAT in the surveys.

**Table:** Table 1

VARIABLE	DEFINITION
Time Categories - Activities included*	
***Market work***	Working in the paid sector, including breaks.
***Unpaid work***	Activities which may be performed by someone else through a salary or a paid service. Made up of *household work* and *purchase of goods*.
*Household work*	Cooking, washing dishes, tiding-up the house, sewing and mending clothes, doing laundry, ironing, dusting, vacuuming, indoor household cleaning, indoor design and maintenance, gardening and pet care, outdoor restructuring and elderly-care.
*Purchase of goods*	Daily grocery shopping, purchase of goods and services for the house and the family members, medical shopping, pet-care items purchase.
***Childcare***	Made up of *Basic childcare* and *Quality childcare*.
*Basic childcare*	Children surveillance, physical and medical care.
*Quality childcare*	Helping children with homework; reading, playing and talking to children.
***Voluntary work***	Helping people outside the family for free, voluntary activities within associations or groups, religious practice and meetings attendance.
***Self-care***	Made up of *sleeping* and *other self-care*.
*Sleeping*	Sleeping.
*Other self-care*	Staying sick in bed, eating and drinking, dressing-up, washing and combing oneself, medical cares.
***Study***	Attending classes, doing homework and studying at any level of education.
**Individual characteristics**	
*Woman*	Dummy variable equal to one if the individual is a woman and zero if the individual is a *man*
*Age range*	Categorical variable specifying to which of the four age categories (25–34, 35–44, 45–54 and 55–64 years) the individual belongs to.
*University*	Dummy variable equal to one if the individual has a university degree and zero if the individual has high school or inferior degree.
*South*	Dummy variable equal to one if the individual lives in the South of Italy or in the islands; zero otherwise.
*Children # y.o.*	Number of children # years old (3–5, 6–10,11–14).
*Married*	Dummy variable equal to one if the individual is married or lives with a partner.
*Employed*	Dummy variable equal to one if the individual is employed.
*Part-time*	Dummy variable equal to one if the individual works part-time.
*Weekend*	Dummy variable equal to one if the individual was surveyed on a weekend day.
**Sector of employment** **	
*Agriculture*	Agriculture, fishing, hunting
*Industry*	Extraction and energy, industry and manufacturing, retail and wholesale trade
*Constructions*	Constructions
*Services*	Hotels and restaurants, transportation, storage and communications, financial intermediation, real estate, renting services, informatics, research and other business activities, public administration and defense, health and other social services.

* Hours per week. All activities include also commuting time devoted to each of them

** The subdivision in four classes for the sector of employment variable was made necessary to make the notation consistent across the three waves of survey. In particular, the 13 categories available for the waves 2002 and 2008 were grouped in 4 categories following the codification available for the wave 2014

We initially dropped from our sample individuals who did not complete the diary (or who did not complete it covering the whole 24-hours-spectrum) and the ones with missing values in variables we use in the analysis (e.g. geographical area of residence or marital status). Since our aim is to quantify gender gaps for individuals that have heavy family duties and share similar opportunity costs in the use of daily time, we selected in the sample of analysis individuals aged 25–54 (ruling out periods in which individuals are in education or retirement), living in a couple (married or cohabitating), where both partners are employed in a full-time job, and having at least one child below age 14.^6^ The resulting sample of full-time working parents (N = 5,778) contains 2,889 women and 2,889 men. The number of observations drops over time across the three survey years by demographic trends and couples’ decisions, moving from 2456 to 2002 to 1458 in 2014 (see the bottom part of Table A1.2 in Appendix 1). Descriptive statistics of hours per week spent in different time categories, by survey year and gender are documented in Table 2a. This table shows that in all waves women spend more time than men in *Household work* and *Basic childcare*, and less time than men in *Market Work*. Starting from 2008, women spend less time than men also in *Quality childcare*. We investigate the magnitude of these gender gaps and their patterns across time through descriptive OLS regressions in the following section.

**Table 2a:** **Descriptive Statistics- Time categories (hours per week) by gender and years**

	Men		Women	
	Mean	sd	Mean	sd
Market work				
2002	36.78	33.92	24.66	28.49
2008	36.98	34.41	25.85	29.19
2014	31.33	33.70	24.76	29.34
Household work				
2002	7.05	11.31	25.89	15.19
2008	8.11	11.58	23.73	14.75
2014	8.55	11.16	22.76	14.53
Basic child				
2002	3.38	6.88	7.66	10.20
2008	3.25	5.77	8.11	10.41
2014	4.58	7.57	8.35	11.18
Quality child				
2002	2.99	5.38	3.35	5.65
2008	3.39	5.65	3.30	5.72
2014	4.23	7.02	3.90	5.76
Leisure				
2002	32.78	21.26	21.48	16.67
2008	33.07	22.38	22.94	17.10
2014	33.31	20.78	23.58	17.39
Obs.	2889		2889	

**Table 2b:** **Descriptive Statistics- Covariates. Years 2002, 2008, 2014 pooled**

Variable	Mean	sd
Woman	0.50	0.50
Age Range 25–34	0.19	0.39
Age Range 35–44	0.56	0.50
Age Range 45–54	0.25	0.43
University	0.21	0.41
South	0.33	0.47
Number of Children	1.46	0.60
Children 0–2 y.o.	0.26	0.47
Children 3–5 y.o.	0.30	0.49
Children 6–10 y.o.	0.42	0.58
Children 11–14 y.o.	0.48	0.60
Weekend	0.63	0.48
Agriculture	0.05	0.22
Industry	0.30	0.46
Construction	0.06	0.23
Services	0.60	0.49
Obs.	5778	

## Empirical Analysis and Results

### OLS Estimates of Gender gaps 

The very simple model we specify by way of appropriate year dummies is:

1 $${Y}^{j}={\delta }_{0}^{j}+{\delta }_{1}^{j}F+{\delta }_{2}^{j}d08+{\delta }_{3}^{j}F*d08+{\delta }_{4}^{j}d14+{\delta }_{5}^{j}F*d14+{\delta }_{6}^{j}X+{u}^{j}$$

where ${Y}^{j}$ measures hours spent in time category *j*, $F$ is a dummy capturing whether the individual is female, *d08, d14* are year dummies and the benchmark group is men belonging to the survey from 2002. Control variables *X* include the following individual characteristics: age, education level, geographical area of residence, number if children, number of children in different age ranges, sector of employment. More precisely, we consider three age groups categories (25–34, 35–44, 45–54), two education categories (high school or less, university or more), two geographical categories (North and South), four categories corresponding to the age of the children within the household (number of children in the age range 0–2, 3–5, 6–10, 11–14, which refer to the type of school they possibly attend - daycare, kindergarten, primary school and middle school), sector of employment (agriculture, industry, constructions, services). Descriptive statistics of all control variables we use in the empirical analysis are available in Table 2b.

We compare figures derived in this sample of full-time working parents aged 25–54 to the ones obtained from a sample of couples aged 25–54 with children below 14 in which only men are employed (N = 6,456); we call it the sample of “traditional couples” and display its results in Appendix 2.^7^

The gender gap “Female-Males” in time use in category *j* in 2002, 2008, 2014 corresponds to the following function of the coefficients: ${\delta }_{1}^{j},{{\delta }_{1}^{j}+{\delta }_{3}^{j},\delta }_{1}^{j}+{\delta }_{5}^{j},$ respectively and can be estimated through OLS. More specifically, we estimate two versions of the gap: without controls (model 1) and with controls (model 2).

For each time use category we first estimate regressions corresponding to model 1 and 2 pooling weekday and weekend day diaries, obtaining results on gender gaps in hours per week in the three years of observations. The results on the coefficients of the control variables are contained in Table A1.5 in Appendix 1. We next estimate interacted versions of model 2 to allow for heterogeneous gender gaps across: (i) the two education categories; (ii) the three age classes. Finally, we estimate separate gender gaps in hours per day for weekdays and weekend days, to account for different time constraints characterizing the two types of day.^8^ The specification including the interaction between year of observation and age class allows us for evaluation of cohort effects in gender gaps, i.e. changes that characterizes individuals born in particular decades, and are independent of the process of aging. For example, comparing individuals aged 25–34 observed in 2014 with individuals of the same age group observed in 2002 we can compare gender gaps at around age 30 for individuals born in the more recent decade (1980–1989) with gender gaps at same age for individuals born about one decade before (1968–1977).

### Patterns in Gender gaps

From Table 2a we can infer trends by gender for the time categories of main interest measured in hours per week. Time spent in *Market work* decreases while female’s one is basically constant. This implies that the gap in *Market work* is falling in the period under study. Similarly, men’s *Household work* slightly increased while female’s decreased; hence also the gap in *Household work* is decreasing during 2002–2014. As for the two types of *Childcare* and *Leisure*, we observe that time devoted to these activities increased for both male and female partners. Hence, we cannot infer information on trends in gender gaps for those activities from Table 2a and we need further analysis.

We can be more specific by linking these trend figures to the underlying estimates obtained for the different categories with model 1 (without controls) contained in the third column of Tables 3, 4, 5, 6 and 7. One observes that the fall in the gap in *Market work* amounts to one hour from 2002 to 2008 and to almost five hours from 2008 to 2014, showing a total decrease of 46% in thirteen years. However, in 2014 full-time working mothers are still working on average 6.6 h per week less than their partners. The decrease in the gap for *Household work* in thirteen years is 4.6 h per week, corresponding to a reduction of 25% but, in 2014, women still devote to the household 14.2 h per week more than men do. No significant change in the gap in *Basic childcare* is observed: in 2014, mothers still devote 3.8 h per week more than their partners to this activity. The gap in *Quality Childcare* – which is the smallest in absolute value- turned from positive to negative in 2008. In 2002 mothers devoted 0.36 h *more* than fathers to this type of care, while in 2014 the figure is reversed, with mothers dedicating 0.3 h *less* than fathers, (but the gap is significative and equal to 0.5 h only if controls are included). Finally, the negative gap in *Leisure* narrowed to slightly less than one hour from 2002 to 2008 and then remained substantially constant from 2008 to 2014. Thus, the gap in *Leisure* decreased by only 1.6 h per week in thirteen years and, in 2014, women still enjoy 9.7 h per week of leisure less than their partners. To sum up, we can observe that all gaps except the one in *Basic childcare* are reducing, but the gap in *Market work* is closing much faster than the others and the gap in *Household work* still amounts to about 14 h per week.

Looking at the last column of Tables 3, 4, 5, 6 and 7 we can uncover cohort effects in gender gaps, comparing results obtained in 2002 and 2014 for individuals in the same age class. More specifically, lets’ call the cohort born in (1980–1989) the *youngest cohort*, the cohort born in (1968–1977) the *young cohort*, that born in (1960–1969) the *old* cohort and that born in (1948–1957) the *oldest cohort*.^9^ The cohort effects in gender gaps are displayed in the “Change 2014 − 2002” section of Tables 3, 4, 5, 6 and 7 where the three figures corresponding to the three age classes compare respectively (i) the *youngest cohort* aged around 30 (observed in 2014) with the *young cohort* at the same age (observed in 2002); (ii) the *young cohort* aged around 40 (observed in 2014) with the *old cohort* at the same age (observed in 2002); (iii) the *old cohort* aged around 50 (observed in 2014) with the *oldest cohort* at the same age (observed in 2002). It is interesting to notice that almost all cohort effects have the expected sign showing smaller (in absolute value) gender gaps for cohorts born in most recent periods, and therefore including individuals grown in a less traditional society. In *Market work*, the highest cohort effect/reduction of the gap is observed between the *youngest* and the *young* cohort at age 30. This can be explained by the fact that in the time span 2002–2014 young Italian women increased their hours of market work while their male counterparts decreased it (see Table A1.4 in Appendix 1). The cohort effects in *Household work* are quite similar to each other at all ages, denoting a quite high persistence of the unbalance in this type of activities across genders. The only statistically significant cohort effect in childcare activities is observed at around age 40 in *Quality Childcare*, testifying the higher involvement of fathers of the *young cohort* with respect to their counterparts of the *old cohort* (born about 10 years before). Finally, a statistically significant positive cohort effect is found for the gender gap in *Leisure* at age 40, signaling a more equal distribution of this category of time across mothers and fathers of the younger generations.

Having depicted the above general patterns emerging from our analysis, in the next subsection we will consider each time category in more detail, with a focus on the different allocation of time occurring during weekdays and weekend days.

### Market Work

We observe a decrease in men’s *Market work* that is aligned with a general trend documented in many other developed countries starting from the 70s, which is mainly driven by low-educated adults; see, among others, Gimenez-Nadal and Sevilla (2012). Specifically, Tables 3, 4, 5, 6 and 7 show that, in Italy, the reduced time that full-time working fathers devote to *Market work* in 2014 with respect to 2002 (-5.5 h per week) is almost compensated by the additional time devoted to *Household work* (+ 1.5 h per week), *Childcare* (+ 2.4 h per week) and *Leisure* (+ 0.5 h per week). Interestingly, the fall in men’s *Market Work* is much more pronounced than in the sample of traditional couples where only male partners are employed (see Table A2.1 in Appendix 2) and amounts to 5.4 h against 3.8. Probably these results depend on the stronger budget pressures existing in traditional families.

**Table 3:** **OLS results - Gender gap in Market work**

Hours per Week											
**Year**	**Male Average**	**Gender Gap** **(Female-Male)**				**University** **degree**			**Age class**		
2002	36.78	-12.122^***^		-13.003^***^		Yes	-14.586^***^		25–34		-16.506^***^
		(0.864)		(0.923)			(2.706)				(2.434)
						No	-12.767^***^		35–44		-12.322^***^
							(1.038)				(1.297)
									45–54		-12.061^***^
											(2.646)
2008	36.98	-11.132^***^		-11.618^***^		Yes	-12.705^***^		25–34		-22.179^***^
		(1.018)		(1.066)			(2.589)				(3.468)
						No	-11.387^***^		35–44		-10.317^***^
							(1.271)				(1.550)
									45–54		-7.145^***^
											(2.260)
2014	31.33	-6.573^***^		-7.163^***^		Yes	-9.574^***^		25–34		-5.504
		(1.088)		(1.154)			(2.207)				(4.350)
						No	-5.782^***^		35–44		-7.725^***^
							(1.545)				(1.749)
									45–54		-6.888^***^
											(2.323)
Change 2014 − 2002		5.549^***^		5.840^***^		Yes	5.013		25–34		11.002^**^
		(1.389)		(1.398)			(3.468)				(4.968)
						No	6.985^***^		35–44		4.597^**^
							(1.790)				(2.150)
									45–54		5.173
											(3.503)
Controls		NO		YES			YES				YES
Obs.		5778		5778			5778				5778
**Hours per day**											
			**Weekday**					**Weekend**			
**Year**			**Male Average**		**Gender Gap** **(Female-Male)**			**Male Average**		**Gender Gap** **(Female-Male)**	
2002			9.07		-2.894^***^			3.08		-1.069^***^	
					(0.206)			3.14		(0.149)	
2008			8.77		-2.375^***^					-1.107^***^	
					(0.224)			2.38		(0.188)	
2014			8.36		-1.598^***^					-0.584^***^	
					(0.264)					(0.191)	
Change 2014 − 2002					1.296^***^					0.484^**^	
					(0.335)					(0.242)	
Controls					NO					NO	
Obs.					2,112					3,666	

Sample of full-time working parents selected from the ISTAT “Indagine Multiscopo sulle famiglie – Uso del tempo”, years 2002, 2008, 2013. **Top panel – Hours per week**. Observations on weekdays and weekend days diaries are pooled. The dependent variable is the weekly time in hours obtained multiplying the observed daily time by 7. Column (3): gender gaps estimated from Eq. (1) without controls. Column (4): gender gaps estimated controlling for: age class, education level, geographical area of residence, number if children, number of children in different age ranges, sector of employment. Column (5): gender gaps estimated by educational level, interacting the female and year dummies in Eq. (1) by a dummy indicating university degree. Column (6): gender gaps estimated by age class, interacting the female and year dummies in Eq. (1) with a set of age classes dummies. **Bottom panel – Hours per day.** Observations on weekdays and weekend days diaries analysed separately. The dependent variable is the daily time in hours. Gender gaps estimated from Eq. (1) without controls. Standard errors are clustered at the family level. *significant a 10%, **significant at 5%, ***significant at 1%.

Despite the gender gap in *Market work* having fallen by 46% in thirteen years, in 2014 full-time working mothers were working 6.6 h (21%) less than full-time working fathers. The persistence of this gap in the sample of full-time working parents may appear surprising at a first glance but it is related to women’s sorting in different sectors of the labor market and different job occupations. Indeed, female workers typically enter less remunerated jobs that are relatively less demanding in terms of working hours, like teaching in elementary, middle, and high schools for example.

Looking at Table 3, one can check that adding controls does not change the results about the gender gap in *Market work* substantially.

Disaggregating the gender gap by education (see the fourth column in Table 3), we observe that the gap in Market work is slightly lower for parents without a university degree than for highly educated parents in 2002 and 2008, while, in 2014 the gap for lower-educated parents is substantially lower than that for highly educated parents. This result is given by the fact that parents with a university degree work systematically fewer hours irrespective of gender than parents without a degree. But, while all fathers and highly-educated mothers decreased time devoted to *Market work*, lower-educated mothers increased their *Market work* in the years under study (see Table A1.3 in Appendix 1).

In the bottom part of Table 3 we display time devoted to *Market Work* during a weekday and during one day of the weekend (either Saturday or Sunday) and we only consider the model without controls given that adding covariates does not alter substantially the results. Disaggregated data show that in 2014 women devote to *Market work* 1.6 h per day (19%) less than men on weekdays and 35 min (24%) less than men on weekend days.

### Household Work

Table 4 shows that the average amount of hours per week of *Household work* provided by men in 2014 amounts to 8.5 h against 22.7 provided by their partners. Hence, women devote to this activity 165% of the time more than men. Again, controls do not affect results substantially.

Interestingly, moving to traditional families (see Table A2.2 in Appendix 2), we observe that, in the same year, men devote 5.8 h per week to *Household work* against 34.8 h of their partners. Recalling that average men’s *Market Work* in traditional families amounts to 34.5 h per week, we conclude that men’s *Market Work* meets their partners’ *Household work*. Comparing the two types of families, we observe that full-time working mothers devote to *Household work* 34% less time than housewives: 22.8 h per week against 34.8 h. As for men, surprisingly, partners of full-time working mothers devote only 2.7 h per week more than partners of housewives to this activity. In addition, the fall in the gap in *Household work* in the period 2002–2014 is the very same in the two samples and amounts to 4.6 h per week. The lower gaps in *Household work* in the two samples are mainly driven by the fall in the time women devote to this activity and only to a lower extent by the additional time their partners devote to it.

This figure is important and suggests that partners of full-time working mothers are not much more inclined, on average, to a fair sharing of household duties in the couple than the representative Italian bread-winner men. Recalling results from the previous subsection 4.3 we observe that, while the negative gap in *Market work* for full-time working mothers reduced by 46% in thirteen years, the positive gap in *Household work* decreased by slightly more than the half (i.e. 25%) in the same period.

Let us consider now disaggregated data for days of the week; see the bottom part of Table 4. In 2014, full-time working mothers devote more time to *Household work* during weekends (3.5 h per day) than in weekdays (2.7 h per day). Likewise, men devote to *Household work* 1.5 h per day in a weekend day against 0.7 h per day during weekends. The interpretation is that, due to the lack of time during the busy weekdays, full-time working parents postpone part of home duties to the weekend; especially men whose increase during weekend days is higher. Table 4 also shows that, during weekends, the gender gap in *Household work* for full-time working parents in 2014 amounts to + 2 h per day (140% more for women). In the same year, on weekdays, full-time working mothers spent on this activity + 2 h per day than their partner (264% more). To conclude, the burden of home duties remains thus very unbalanced, mainly on weekdays.

**Table 4:** OLS results - Gender gap in Household work

Hours per Week											
**Year**	**Male Average**	**Gender Gap** **(Female-Male)**				**University** **degree**			**Age class**		
2002	7.05	18.848^***^		19.243^***^		Yes	17.373^***^		25–34		17.487^***^
		(0.497)		(0.512)			(1.244)				(0.972)
						No	19.563^***^		35–44		19.337^***^
							(0.565)				(0.661)
									45–54		22.598^***^
											(1.577)
2008	8.11	15.625^***^		16.049^***^		Yes	14.672^***^		25–34		16.307^***^
		(0.572)		(0.583)			(1.033)				(1.404)
						No	16.416^***^		35–44		14.611^***^
							(0.689)				(0.786)
									45–54		17.938^***^
											(1.283)
2014	8.55	14.208^***^		14.741^***^		Yes	14.591^***^		25–34		12.940^***^
		(0.625)		(0.643)			(1.009)				(1.921)
						No	14.832^***^		35–44		14.304^***^
							(0.813)				(0.850)
									45–54		16.533^***^
											(1.308)
Change 2014 − 2002		-4.640^***^		-4.502^***^		Yes	-2.783^*^		25–34		-4.547^**^
		(0.798)		(0.802)			(1.595)				(2.154)
						No	-4.730^***^		35–44		-5.033^***^
							(0.970)				(1.068)
									45–54		-6.065^***^
											(2.044)
Controls		NO		YES			YES				YES
Obs.		5778		5778			5778				5778
**Hours per day**											
			**Weekday**					**Weekend**			
**Year**			**Male Average**		**Gender Gap** **(Female-Male)**			**Male Average**		**Gender Gap** **(Female-Male)**	
2002			0.61		2.663^***^			1.24		2.710^***^	
					(0.100)					(0.096)	
2008			0.76		2.110^***^			1.40		2.307^***^	
					(0.113)					(0.112)	
2014			0.74		1.957^***^			1.48		2.069^***^	
					(0.127)					(0.119)	
Change 2014 − 2002					-0.706^***^					-0.641^***^	
					(0.162)					(0.153)	
Controls					NO					NO	
Obs.					2,112					3,666	

Sample of full-time working parents selected from the ISTAT “Indagine Multiscopo sulle famiglie – Uso del tempo”, years 2002, 2008, 2013. **Top panel – Hours per week**. Observations on weekdays and weekend days diaries are pooled. The dependent variable is the weekly time in hours obtained multiplying the observed daily time by 7. Column (3): gender gaps estimated from Eq. (1) without controls. Column (4): gender gaps estimated controlling for: age class, education level, geographical area of residence, number if children, number of children in different age ranges, sector of employment. Column (5): gender gaps estimated by educational level, interacting the female and year dummies in Eq. (1) by a dummy indicating university degree. Column (6): gender gaps estimated by age class, interacting the female and year dummies in Eq. (1) with a set of age classes dummies. **Bottom panel – Hours per day.** Observations on weekdays and weekend days diaries analysed separately. The dependent variable is the daily time in hours. Gender gaps estimated from Eq. (1) without controls. Standard errors are clustered at the family level. *significant a 10%, **significant at 5%, ***significant at 1%.

### Childcare

Tables 5 and 6 show data for *Basic childcare* and *Quality childcare*, respectively. Interestingly, men devote about the same amount of time to *Basic childcare* and to *Quality childcare*, women instead devote much more time to *Basic childcare* than to *Quality childcare*. As it will be clear in the following, *Basic childcare* remains mainly a responsibility of mothers while fathers are relatively more involved in *Quality childcare.* Our interpretation is the following. On the one side, *Quality childcare* is a more gratifying way of spending time with children (also because no social stigma for fathers^10^ is associated with this type of activity). On the other side, *Quality childcare* is perceived as more directly related to the development of children’s cognitive skills and school abilities so it has a relatively more tangible return than *Basic childcare*. In addition, the OLS results on the coefficients of the covariate included in Model 2 (reported in Table A1.5 in Appendix 1), show that tertiary education has a positive impact on the provision of both *Basic childcare* and *Quality childcare*, meaning that more educated parents, and especially mothers, provide more informal care.

**Table 5:** OLS results - Gender gap in Basic childcare

Hours per Week											
**Year**	**Male Average**	**Gender Gap** **(Female-Male)**				**University** **degree**			**Age class**		
2002	3.38	4.274^***^		4.159^***^		Yes	5.719^***^		25–34		7.625^***^
		(0.290)		(0.299)			(0.990)				(0.703)
						No	3.883^***^		35–44		3.837^***^
							(0.319)				(0.379)
									45–54		1.086^*^
											(0.624)
2008	3.25	4.861^***^		4.661^***^		Yes	6.816^***^		25–34		9.154^***^
		(0.355)		(0.359)			(0.791)				(1.007)
						No	4.061^***^		35–44		4.795^***^
							(0.393)				(0.465)
									45–54		1.311^***^
											(0.479)
2014	4.58	3.769^***^		3.556^***^		Yes	5.519^***^		25–34		5.893^***^
		(0.437)		(0.440)			(0.864)				(1.238)
						No	2.706^***^		35–44		4.256^***^
							(0.489)				(0.635)
									45–54		0.898
											(0.594)
Change 2014 − 2002		-0.505		-0.604		Yes	-0.200		25–34		-1.732
		(0.524)		(0.520)			(1.318)				(1.416)
						No	-1.176^**^		35–44		0.419
							(0.569)				(0.734)
									45–54		-0.188
											(0.854)
Controls		NO		YES			YES				YES
Obs.		5778		5778			5778				5778
**Hours per day**											
			**Weekday**					**Weekend**			
**Year**			**Male Average**		**Gender Gap** **(Female-Male)**			**Male Average**		**Gender Gap** **(Female-Male)**	
2002			0.42		0.775^***^			0.52		0.517^***^	
					(0.069)					(0.052)	
2008			0.49		0.871^***^			0.45		0.586^***^	
					(0.088)					(0.061)	
2014			0.49		0.615^***^			0.74		0.497^***^	
					(0.100)					(0.080)	
Change 2014 − 2002					-0.159					-0.020	
					(0.121)					(0.095)	
Controls					NO					NO	
Obs.					2,112					3,666	

Sample of full-time working parents selected from the ISTAT “Indagine Multiscopo sulle famiglie – Uso del tempo”, years 2002, 2008, 2013. **Top panel – Hours per week**. Observations on weekdays and weekend days diaries are pooled. The dependent variable is the weekly time in hours obtained multiplying the observed daily time by 7. Column (3): gender gaps estimated from Eq. (1) without controls. Column (4): gender gaps estimated controlling for: age class, education level, geographical area of residence, number if children, number of children in different age ranges, sector of employment. Column (5): gender gaps estimated by educational level, interacting the female and year dummies in Eq. (1) by a dummy indicating university degree. Column (6): gender gaps estimated by age class, interacting the female and year dummies in Eq. (1) with a set of age classes dummies. **Bottom panel – Hours per day.** Observations on weekdays and weekend days diaries analysed separately. The dependent variable is the daily time in hours. Gender gaps estimated from Eq. (1) without controls. Standard errors are clustered at the family level. *significant a 10%, **significant at 5%, ***significant at 1%.

**Table 6:** OLS results - Gender gap in Quality childcare

Hours per Week											
**Year**	**Male Average**	**Gender Gap** **(Female-Male)**				**University** **degree**			**Age class**		
2002	2.99	0.357^*^		0.253		Yes	0.419		25–34		-0.111
		(0.185)		(0.197)			(0.653)				(0.455)
						No	0.222		35–44		0.411
							(0.205)				(0.268)
									45–54		0.685
											(0.451)
2008	3.39	-0.088		-0.187		Yes	-0.032		25–34		-0.302
		(0.210)		(0.215)			(0.492)				(0.678)
						No	-0.217		35–44		-0.368
							(0.251)				(0.316)
									45–54		0.329
											(0.352)
2014	4.23	-0.330		-0.510^*^		Yes	-0.482		25–34		-0.190
		(0.287)		(0.292)			(0.616)				(1.114)
						No	-0.525		35–44		-1.111^***^
							(0.342)				(0.428)
									45–54		0.127
											(0.434)
Change 2014 − 2002		-0.688^**^		-0.763^**^		Yes	-0.901		25–34		-0.079
		(0.341)		(0.344)			(0.897)				(1.204)
						No	-0.747^*^		35–44		-1.523^***^
							(0.390)				(0.504)
									45–54		-0.558
											(0.621)
Controls		NO		YES			YES				YES
Obs.		5778		5778			5778				5778
**Hours per day**											
			**Weekday**					**Weekend**			
**Year**			**Male Average**		**Gender Gap** **(Female-Male)**			**Male Average**		**Gender Gap** **(Female-Male)**	
2002			0.38		0.104^**^			0.46		0.021	
					(0.043)					(0.033)	
2008			0.47		0.027			0.49		-0.037	
					(0.048)					(0.039)	
2014			0.39		0.133^**^			0.72		-0.144^***^	
					(0.061)					(0.053)	
Change 2014 − 2002					0.029					-0.165^***^	
					(0.075)					(0.063)	
Controls					NO					NO	
Obs.					2,112					3,666	

Sample of full-time working parents selected from the ISTAT “Indagine Multiscopo sulle famiglie – Uso del tempo”, years 2002, 2008, 2013. **Top panel – Hours per week**. Observations on weekdays and weekend days diaries are pooled. The dependent variable is the weekly time in hours obtained multiplying the observed daily time by 7. Column (3): gender gaps estimated from Eq. (1) without controls. Column (4): gender gaps estimated controlling for: age class, education level, geographical area of residence, number if children, number of children in different age ranges, sector of employment. Column (5): gender gaps estimated by educational level, interacting the female and year dummies in Eq. (1) by a dummy indicating university degree. Column (6): gender gaps estimated by age class, interacting the female and year dummies in Eq. (1) with a set of age classes dummies. **Bottom panel – Hours per day.** Observations on weekdays and weekend days diaries analysed separately. The dependent variable is the daily time in hours. Gender gaps estimated from Eq. (1) without controls. Standard errors are clustered at the family level. *significant a 10%, **significant at 5%, ***significant at 1%.

Let us first focus on *Basic childcare* and Table 5. In the thirteen years under study both full time-working parents increased the time devoted to *Basic care*. In 2014 fathers devoted to *Basic childcare* 4.6 h per week and women 8.3 h, which means that mothers devoted to this activity almost 82% more time than their partners. Looking at the per-week time devoted to *Basic childcare*, the gender gap remains stable across the thirteen years. Interestingly, the gender gap in *Basic childcare* is much higher among individuals holding a university degree, the reason being that more-educated mothers devote more time to informal childcare than low-educated ones. Indeed, while the gender gap in time devoted to *Basic childcare* among parents with a university degree did not change, the gap among low-educated couples falls of 1.2 h per week in the period under study.

As expected, partners of full-time working mothers devote more time to Basic childcare than fathers in traditional families: 4.6 h per week against 2.9 in 2014. And the gender gap in this time category is 3.8 h per week in modern families against 7.5 h in traditional families.

Looking at disaggregated figures, full-time working mothers devoted to *Basic childcare* 36 min more per day than their partners during weekdays in 2014; whereas they devoted 46 min more per day in 2002. In 2014, the gender gap in time devoted to *Basic childcare* is slightly lower in weekend days than in days of the week (30 min against 36).

Moving to *Quality care* and checking Table 6, we observe many interesting phenomena. First, in the thirteen years under study, fathers almost doubled the time they devote to *Quality childcare*. In 2014, fathers spend 4.2 h per week in this activity against slightly less than 4 h of their partners. The gender gap was positive in 2002 and equal to about 22 min per week but it changed its sign starting from the 2008’s wave and Italian full-time working fathers now devote more time to *Quality childcare* than full-time working mothers. Specifically, the negative gender gap is significant only when we include controls and amounts to 30 min per week, corresponding to a percentage value of -12%. For the sake of comparison, in traditional families, fathers devoted 3.3 h per week and their partners 1.4 h more to *Quality childcare* in 2014.

Interestingly, looking at disaggregated data for full-time working parents in the bottom part of Table 6, we observe that the gender gap is still positive in weekdays, but it is negative in weekends. Then we conclude that the change in the sign of the gender gap for *Quality childcare* is driven by fathers’ behaviors in weekends. Specifically, in 2014, fathers devoted 23.4 min per day to *Quality childcare* in weekdays, against 31.4 min of their partners, with a positive gender gap of 8 min per day, meaning that full-time working mothers dedicate to this activity the 33% more of the time than their partners. During weekend days, instead, fathers devote 43.2 min per day to *Quality childcare* against 34,6 min per day of their partners, with a negative gap of about 9 min per day.

To sum up both fathers and mothers increased the time they devote to the two types of childcare in the thirteen years under study (see Table 2a). This corresponds to a general trend in developed countries that is particularly evident for younger and more educated people. The evolution of the gap in the two types of childcare activities is however different. Despite working full-time like their partners, mothers still maintain the main responsibility of both types of childcare during weekdays. Instead, during weekend days, their partners become the main provider of *Quality childcare* thus reversing the gender gap for this specific informal care activity.

As a final observation, the share of family duties during weekend days for full-time working partners is more balanced. Conversely, in traditional families, the positive gender gap in *Quality childcare* is persisting (no changes are observed in the period under study) and amounts to 1.4 h per week in 2014. Hence, when women are not employed, fathers’ engagement in both types of childcare remains much lower.

### Leisure

Table 2a shows that fathers’ and especially mothers’ *Leisure* increased in the three surveys. As a result, the gender gap in *Leisure* falls in the thirteen years under study; see the upper part of Table 7. Importantly, this result is fully driven by parents without a university degree. Indeed, we observe that the gender gap in *Leisure* increases in the period under study among parents with a university degree and amounts to -11.2 h per week in 2014, while it was only − 7.2 in 2002. Hence, while in 2002 the gap in *Leisure* was larger among parents without a university degree, in 2014 it is larger among parents with a university degree. The reason being that mothers with a university degree tend to provide more *Market work* and more *Basic childcare* than mothers without a university degree. The latter instead provide more *Household work* than more educated mothers, but not enough to fully compensate for the additional time devoted to the other time categories by women with a university degree.

Comparing modern and traditional families, it is interesting to remark that partners of full-time working mothers enjoy one hour of leisure less per week than partners of unemployed mothers in 2014. In addition, the gender gap in *Leisure* amounts to -9.7 h per week in modern families and to -6.9 in traditional families. Hence, full-time working mothers benefit from about 4 h per week of *Leisure* less than their counterparts in traditional families. Among full-time working parents, thus, gender inequality in *Leisure* is larger than in traditional couples. This confirms that, in a country with conservative gender attitudes like Italy, the period in which children are young is extremely demanding for full-time working mothers.

Looking more in detail at the gender gap in the couple, in 2014 fathers took advantage of 33.3 h per week of *Leisure* while women of only 23.6 with a gap of -9.7 h, amounting to the 29% less of the time that men devoted to this activity.

**Table 7:** OLS results - Gender gap in Leisure

Hours per Week											
**Year**	**Male Average**	**Gender Gap** **(Female-Male)**				**University** **degree**			**Age class**		
2002	32.78	-11.301^***^		-10.707^***^		Yes	-7.207^***^		25–34		-7.621^***^
		(0.532)		(0.563)			(1.917)				(1.372)
						No	-11.271^***^		35–44		-12.217^***^
							(0.620)				(0.810)
									45–54		-10.465^***^
											(1.654)
2008	33.07	-10.130^***^		-9.743^***^		Yes	-8.559^***^		25–34		-4.472^**^
		(0.622)		(0.651)			(1.627)				(1.987)
						No	-9.967^***^		35–44		-9.270^***^
							(0.781)				(0.930)
									45–54		-13.409^***^
											(1.505)
2014	33.31	-9.732^***^		-9.326^***^		Yes	-11.225^***^		25–34		-8.721^***^
		(0.691)		(0.730)			(1.578)				(2.236)
						No	-8.787^***^		35–44		-9.295^***^
							(0.916)				(1.119)
									45–54		-9.432^***^
											(1.571)
Change 2014 − 2002		1.570^*^		1.381		Yes	-4.017		25–34		-1.100
		(0.873)		(0.878)			(2.472)				(2.614)
						No	2.484^**^		35–44		2.922^**^
							(1.065)				(1.372)
									45–54		1.032
											(2.268)
Controls		NO		YES			YES				YES
Obs.		5778		5778			5778				5778
**Hours per day**											
			**Weekday**					**Weekend**			
**Year**			**Male Average**		**Gender Gap** **(Female-Male)**			**Male Average**		**Gender Gap** **(Female-Male)**	
2002			2.93		-0.839^***^			5.68		-2.057^***^	
					(0.106)					(0.100)	
2008			3.00		-0.934^***^			5.78		-1.763^***^	
					(0.124)					(0.120)	
2014			3.28		-1.004^***^			5.56		-1.598^***^	
					(0.147)					(0.129)	
Change 2014 − 2002					-0.164					0.458^***^	
					(0.181)					(0.163)	
Controls					NO					NO	
Obs.					2,112					3,666	

Sample of full-time working parents selected from the ISTAT “Indagine Multiscopo sulle famiglie – Uso del tempo”, years 2002, 2008, 2013. **Top panel – Hours per week**. Observations on weekdays and weekend days diaries are pooled. The dependent variable is the weekly time in hours obtained multiplying the observed daily time by 7. Column (3): gender gaps estimated from Eq. (1) without controls. Column (4): gender gaps estimated controlling for: age class, education level, geographical area of residence, number if children, number of children in different age ranges, sector of employment. Column (5): gender gaps estimated by educational level, interacting the female and year dummies in Eq. (1) by a dummy indicating university degree. Column (6): gender gaps estimated by age class, interacting the female and year dummies in Eq. (1) with a set of age classes dummies. **Bottom panel – Hours per day.** Observations on weekdays and weekend days diaries analysed separately. The dependent variable is the daily time in hours. Gender gaps estimated from Eq. (1) without controls. Standard errors are clustered at the family level. *significant a 10%, **significant at 5%, ***significant at 1%.

Here again, disaggregating data on weekdays and weekends provides interesting insights. The bottom part of Table 7 shows that, on weekdays, the gender gap remains constant for period under study. Specifically, in 2014, fathers took advantage of 3.3 h per day of *Leisure* in weekdays against the 2.3 h of their partners with a gap of 1 h per day. In weekend days we observe a larger gap: fathers took advantage of 5.6 h per day of *Leisure* against slightly less than 4 h per day of their partners, with a gap of -1.6 h per day. This amounts to a gender gap of 29% in favor of fathers.

To sum up, the gender gap in *Leisure* remained constant on weekdays where, despite working full-time as their male partners, women devote to *Leisure* 1 h per day less than their partners. In addition, on weekdays the gender gap in *Leisure* remained stable during the thirteen years under study meaning that we do not observe any improvement in this respect. The situation is becoming slightly more balanced on weekend days because here the gender gap fell of 22% in the period under study.

## Conclusion

This work provides novel evidence on the entity of the gender gap in time use and its evolution in the period 2002–2014 for Italian couples with young children. Exploiting data from three Time Use Surveys from the Italian National Institute of Statistics (ISTAT), including the most recent one available, we provide an up-to-date picture of the time parents devote to the main daily activities: *Market work, Household work, Childcare* and *Leisure*.

We show that, the gender gap in *Market work* is closing fast and in weekdays full-time working mothers are working only the 19% less than their partners (-1.6 h per day) in 2014. However, family duties remain a female responsibility, mainly during weekdays. Specifically, in weekdays full-time working mothers still devote 264% more time to *Household work* (+ 2 h per day), 125% more time to *Basic childcare* (+ 0.6 h per day) and 33% more time to *Quality childcare* (+ 0.13 h per day) than full-time working fathers, which translates in 30% less time (-1 h per weekday) of *Leisure* for mothers. A higher relative contribution to household duties is given by male partners during weekend days. Nevertheless, also during weekend days gender imbalance is important and full-time working mothers still provide 140% more time to *Household work* (+ 2 h per day) and experience 29% less time of *Leisure* than their partners (-1.6 h per day).

Our results thus describe a very unbalanced picture of Italian families in the first two decades of the 21st century: in 2014 the total amount of formal and informal work (*Market work, Household work* and *Childcare*) of full-time employed mothers with young children is about 60 h per week (25 h of *Market work* plus 35 h of *Household work*, *Basic and Quality childcare*) against the 49 h provided by their male partners. This translates into a gender gap in formal and informal work of 11 h per week which is largely superior to the European average.^11^ Partners of full-time working mothers are not more inclined, on average, to a fair sharing of domestic chores in the couple than bread-winner men belonging to the sample of families where women are not employed. Specifically, partners of full-time working mothers only spend 2.7 h per week more on *Household work* than their bread-winner counterparts and only 3 h per week more on childcare. Italian conservative gender attitudes might contribute to explaining why the recent increased involvement of women in the labor market is still far from being compensated by a sufficiently larger involvement of men in the family and household duties.

On the positive side, we document a positive trend in time spent by fathers in *Childcare* and the reversal of the sign of the gender gap in *Quality childcare* during weekend days. However, fathers still maintain a different attitude towards *Basic childcare* and *Quality childcare.* Together with being a less “female-typed” activity, *Quality childcare* is probably perceived as more gratifying and more psychologically rewarding.

Increasing the take-up of parental leave by fathers could contribute to enhancing fathers’ involvement in *Basic childcare*. Indeed, Tamm (2019) shows that even short periods of paternity leave may have long-lasting effects on fathers’ involvement in childcare and housework and that effects on maternal labor supply are also significantly positive (but do not persist over time).^12^ To comply with European directives, the Italian Budget Law for the year 2021 (Law 30 December 2020, No. 178) introduced an increase of the mandatory government-paid paternity leave from seven working days to ten working days. This change goes in the right direction. However, despite the benefit being mandatory, there are no specific penalties associated with non-compliance. As an example, in 2018, when the length of mandatory paternity leave was still seven working days, its take-up has been only the 48% of mandatory maternity leave, showing that cultural resistance to fathers’ *Basic childcare* provision is still persisting in Italy (Casarico and Kopiska 2020).

While the gender gap in parental leave take-up is currently debated by the media and by politicians in Italy, unfortunately, a public debate on gender balance in housework is fully missing. Probably, housekeeping is associated with “low status” and a “negative stigma” even among partners of full-time working women. And policies able to encourage an equal share of domestic chores inside the household are not easy to design. For example, a subsidy on formal domestic work would alleviate the current burden of working mothers. However, this would represent a regressive policy and the richer households would benefit from the policy relatively more.^13^

Our evidence suggests that women’s participation in the labor market and the rise of woman’s bargaining power inside the couple are far from being sufficient to ensure gender equality inside the household.

## Electronic Supplementary Material

Below is the link to the electronic supplementary material.

Supplementary Material 1

Supplementary Material 2

## Data Availability

Data are provided by the Italian Statistical Institute and must be requested for research purposes.

## Footnotes

1 The OECD (2019) Gender Data Portal reports figures elaborated from the most recent waves of time use surveys of the different countries. The time spent daily in unpaid work by women aged 15–64 amounts to 306 min for Italy against the 262,4 min of the OECD average. Italy ranks 4th after The Netherland (331 min), Portugal (328) and Australia (311).

2 However, considering labor supply at the extensive margin, the participation rate of highly-educated women remains higher than the one of low-educated women. Quoting Bettio and Pastore (2017, page 4): “Well educated women are much more likely to work and to work without long interruptions to care for children or relatives. Poorly educated women are much less likely to be in employment especially in the Mezzogiorno. This difference between well and poorly educated women is to be found in most countries across the world, but what distinguishes Italy and other Southern European countries from the rest of Europe is that poorly educated women are comparatively more likely not to work and not to return to the labour after marriage (or childbearing).”

3 Percentages are computed by considering the ratio (women’s time – men’s time)/men’s time. The time is either considered per week or per day, according to which one is relevant.

4 This simplifying choice makes our results comparable to those of studies using MTUS data, such as Gimenez-Nadal and Sevilla (2012). Folbre and Yoon (2007) stress that when only main activities are considered, time in childcare underestimates the total time spent with children, since childcare is sometimes performed as a secondary activity. Accordingly, in our analysis, time devoted to *Basic childcare* is likely to be underestimated. On the contrary, our definition of *Quality childcare* is not compatible with *other* activities being performed simultaneously, and therefore *Quality childcare* is unlikely to be underestimated.

5 In the paper we decided to disregard *Purchase of goods* as a component of *Unpaid work* for two reasons. First, the representative Italian person devotes to *Household work* relatively much more time than to *Purchase of goods* (19 hours per week against 5 hours); second, the gap in *Purchase of goods* remains substantially stable in the thirteen years under study (and amounts to less than + 2 hours per week).

6 We selected out of our sample individuals living in households with more than one family unit and individuals different from the reference individual and his/her partner. We also dropped couples in which only one of the two partners completed the time-use diary.

7 In a working paper version, Barigozzi et al. 2020, we also present the analysis of a complete sample (N = 69,381) containing all individuals aged 25–64. With respect to this complete sample, our sample of full-time working parents contains younger and more educated individuals. Clearly, our sample does not reflect decreasing patterns in fertility and marriage across the available waves. This can be observed by comparing the descriptive statistics on the number of children and marriage by waves contained in Table A2 of the working paper with Table A1.2 of Appendix 1 of the present paper.

8 The results in (ii) and (iii) are obtained splitting the observations between weekday (about one third of the total) and weekend (about two thirds) and without inserting controls, since these turn out to leave the estimated gaps almost unaffected.

9 The youngest cohort is observed only once around age 30 in 2014, the young and old cohorts are observed twice: the young cohort around age 30 in 2002 and around age 40 in 2014; the old cohort around age 40 in 2002 and around age 50 in 2014. The oldest cohort is observed only once around age 50 in 2002.

10 See, as an example, Haas and Hwang (2019) and references within. For a non-academic general overview see Paternity Leave: The Rewards and the Remaining Stigma. The NYT, Nov. 7, 2014; available at https://www.nytimes.com/2014/11/09/upshot/paternity-leave-the-rewards-and-the-remaining-stigma.html.

11 Based on survey data from Special Eurobarometer 465, the 2018 Report on Equality Between Women and Men in the EU indicates that, in couples with the youngest child under 7, women spend on average 32 h per week on *Market work* but 39 h on *Household work* and *Childcare*, conversely men spend 41 h in paid work and 19 h in *Household work* and *Childcare* per week. In our subsample of full-time working parents with at least one child under 14, women provide less *Market work* and less *Household work* + *Childcare* than the EU average but the gender gap in total work is higher in Italy than in the average of European countries (11 against 9 h per week on average in the EU).

12 Paternity leave might also have some unintended consequences. Farré and González (2019) find that the introduction of paid paternity leave led to delays in subsequent fertility. Specifically, parents entitled to the new paternity leave took longer to have another child compared to ineligible parents and, older eligible couples were less likely to have an additional child after the introduction of the reform. Two potentially complementary channels could explain those negative effects. First, fathers’ increasing involvement in childcare led to higher labor force attachment among mothers. This may have raised the opportunity cost of an additional child. Moreover, men reported lower desired fertility after the reform, possibly due to their increased awareness of the costs of childrearing, or to a shift in preferences from child quantity to quality.

13 In the seventies, the Italian movement “Wages for Housework” received some echoes and, in 2014, the idea that the government (or, as an alternative, the husband) should pay women for domestic work reached again the political debate. The advocates of the allowance for housewives think that domestic work should be rewarded to let housewives be economically independent. Such a policy, however, would reinforce the social norm about domestic work as a “women’s work” and, in addition, would discourage women’s participation in the labor market.
