# Supplementary material for: The Gender Gaps in Time-Use Within Italian Households During 2002–2014
Source: Ital Econ J. 2022 Oct 21:1–34. Online ahead of print. doi: 10.1007/s40797-022-00211-5 (PMC9589588; doi:10.1007/s40797-022-00211-5)
Supplement: Supplementary file 1 — Supplementary Material 1 [file 40797_2022_211_MOESM1_ESM.docx]

Appendix 1

**Table A1.1 -Description of ISTAT time-use surveys**

| **SURVEY NAME** | **SURVEY COVERAGE** | **SAMPLE STRUCTURE** | **SIZE** | **ACTIVITIES** |
| --- | --- | --- | --- | --- |
| Istat Uso del Tempo | Apr.2002- Mar. 2003 | Individuals aged 3 years old and over (we select those in the age range 25-64). The activities are recorded by the individual using a diary form survey, both on working days and on weekends. Intervals are of 10 minutes, with a total of 144 records for each diary. We only consider primary activity. | 51,206 | 262 |
| Istat Uso del Tempo | Feb. 2008-Jan. 2009 | Individuals aged 3 years old and over (we select those in the age range 25-64). The activities are recorded by the individual using a diary form survey, both on working days and on weekends. Intervals are of 10 minutes, with a total of 144 records for each diary. We only consider primary activity. | 40,944 | 258 |
| Istat Uso del Tempo | Nov. 2013-Oct. 2014 | Individuals aged 3 years old and over (we select those in the age range 25-64). The activities are recorded by the individual using a diary form survey, both on working days and on weekends. Intervals are of 10 minutes, with a total of 144 records for each diary. We only consider primary activity. | 41229 | 147 |

#

| **Table A1.2 – Descriptive Statistics – Covariates - by year** | | |
| --- | --- | --- |
| Variable | Mean | sd |
| Woman |  |  |
| 2002 | 0,50 | 0,50 |
| 2008 | 0,50 | 0,50 |
| 2014 | 0,50 | 0,50 |
| Age Range 25-34 |  |  |
| 2002 | 0,23 | 0,42 |
| 2008 | 0,17 | 0,38 |
| 2014 | 0,13 | 0,33 |
| Age Range 35-44 |  |  |
| 2002 | 0,57 | 0,49 |
| 2008 | 0,56 | 0,50 |
| 2014 | 0,54 | 0,50 |
| Age Range 45-54 |  |  |
| 2002 | 0,19 | 0,40 |
| 2008 | 0,27 | 0,44 |
| 2014 | 0,33 | 0,47 |
| University |  |  |
| 2002 | 0,14 | 0,35 |
| 2008 | 0,23 | 0,42 |
| 2014 | 0,30 | 0,46 |
| South |  |  |
| 2002 | 0,33 | 0,47 |
| 2008 | 0,30 | 0,46 |
| 2014 | 0,35 | 0,48 |
| Number of Children |  |  |
| 2002 | 1,47 | 0,61 |
| 2008 | 1,42 | 0,56 |
| 2014 | 1,50 | 0,60 |
| Children 0-2 y.o. |  |  |
| 2002 | 0,27 | 0,47 |
| 2008 | 0,26 | 0,47 |
| 2014 | 0,23 | 0,46 |
| Children 3-5 y.o. |  |  |
| 2002 | 0,27 | 0,48 |
| 2008 | 0,31 | 0,49 |
| 2014 | 0,32 | 0,50 |
| Children 6-10 y.o. |  |  |
| 2002 | 0,40 | 0,57 |
| 2008 | 0,37 | 0,55 |
| 2014 | 0,54 | 0,64 |
| Children 11-14 y.o. |  |  |
| 2002 | 0,54 | 0,62 |
| 2008 | 0,48 | 0,58 |
| 2014 | 0,41 | 0,59 |
| Weekend |  |  |
| 2002 | 0,64 | 0,48 |
| 2008 | 0,62 | 0,49 |
| 2014 | 0,65 | 0,48 |
| Agriculture |  |  |
| 2002 | 0,06 | 0,23 |
| 2008 | 0,05 | 0,21 |
| 2014 | 0,04 | 0,21 |
| Industry |  |  |
| 2002 | 0,35 | 0,48 |
| 2008 | 0,34 | 0,48 |
| 2014 | 0,16 | 0,36 |
| Construction |  |  |
| 2002 | 0,05 | 0,23 |
| 2008 | 0,05 | 0,22 |
| 2014 | 0,06 | 0,24 |
| Services |  |  |
| 2002 | 0,54 | 0,50 |
| 2008 | 0,56 | 0,50 |
| 2014 | 0,74 | 0,44 |
| Obs. |  |  |
| 2002 | 2456 |  |
| 2008 | 1864 |  |
| 2014 | 1458 |  |

**Table A1.3 – Descriptive Statistics –** **Time categories by gender, year and education**

Men Women

Univ No Univ Univ No Univ

|  | Mean | sd | Mean | sd | Mean | sd | Mean | sd |
| --- | --- | --- | --- | --- | --- | --- | --- | --- |
| Market work |  |  |  |  |  |  |  |  |
| 2002 | 33.54 | 32.51 | 37.28 | 34.12 | 19.64 | 26.26 | 25.56 | 28.80 |
| 2008 | 33.20 | 33.73 | 37.88 | 34.53 | 21.55 | 26.82 | 27.40 | 29.86 |
| 2014 | 26.46 | 32.25 | 33.00 | 34.05 | 17.77 | 25.90 | 28.47 | 30.39 |
| Household work |  |  |  |  |  |  |  |  |
| 2002 | 6.62 | 9.47 | 7.11 | 11.57 | 23.44 | 14.24 | 26.34 | 15.32 |
| 2008 | 8.20 | 10.32 | 8.09 | 11.87 | 22.35 | 13.89 | 24.23 | 15.03 |
| 2014 | 7.28 | 8.60 | 8.99 | 11.88 | 21.33 | 13.91 | 23.52 | 14.81 |
| Basic childcare |  |  |  |  |  |  |  |  |
| 2002 | 4.78 | 8.43 | 3.17 | 6.58 | 11.12 | 11.80 | 7.04 | 9.76 |
| 2008 | 3.86 | 5.85 | 3.11 | 5.75 | 10.53 | 11.60 | 7.24 | 9.81 |
| 2014 | 5.32 | 8.36 | 4.33 | 7.28 | 11.05 | 12.95 | 6.92 | 9.84 |
| Quality childcare |  |  |  |  |  |  |  |  |
| 2002 | 3.56 | 6.21 | 2.91 | 5.24 | 4.44 | 6.97 | 3.16 | 5.35 |
| 2008 | 3.73 | 5.68 | 3.30 | 5.65 | 3.78 | 6.13 | 3.12 | 5.55 |
| 2014 | 4.82 | 7.90 | 4.03 | 6.69 | 4.62 | 5.94 | 3.52 | 5.63 |
| Leisure |  |  |  |  |  |  |  |  |
| 2002 | 33.04 | 20.68 | 32.74 | 21.36 | 25.02 | 18.26 | 20.84 | 16.29 |
| 2008 | 33.68 | 22.48 | 32.92 | 22.37 | 24.70 | 17.92 | 22.30 | 16.77 |
| 2014 | 37.92 | 22.70 | 31.73 | 19.85 | 26.00 | 18.28 | 22.29 | 16.78 |
| Obs. | 529 |  | 2360 |  | 687 |  | 2202 |  |

**Table A1.4 – Descriptive Statistics – Time categories by gender, year and age class**

|  |  | | Men | |  | |  | | Women | |  | |
| --- | --- | --- | --- | --- | --- | --- | --- | --- | --- | --- | --- | --- |
|  | Age 25-34 | | Age 35-44 | | Age 45-54 | | Age 25-34 | | Age 35-44 | | Age 45-54 | |
|  | Mean | sd | Mean | sd | Mean | sd | Mean | sd | Mean | sd | Mean | sd |
| Market work |  |  |  |  |  |  |  |  |  |  |  |  |
| 2002 | 37.60 | 35.48 | 38.54 | 34.03 | 32.89 | 32.48 | 22.07 | 28.28 | 26.49 | 28.67 | 21.18 | 27.35 |
| 2008 | 44.24 | 33.96 | 36.72 | 34.25 | 35.10 | 34.60 | 22.37 | 28.58 | 26.59 | 29.27 | 27.89 | 29.49 |
| 2014 | 31.67 | 35.16 | 32.71 | 33.50 | 29.76 | 33.65 | 26.85 | 30.22 | 24.79 | 29.26 | 23.13 | 28.99 |
| Household work |  |  |  |  |  |  |  |  |  |  |  |  |
| 2002 | 5.95 | 10.01 | 6.51 | 10.89 | 8.74 | 12.61 | 23.71 | 13.71 | 26.02 | 15.28 | 31.25 | 17.14 |
| 2008 | 7.32 | 10.28 | 8.13 | 11.83 | 8.32 | 11.60 | 23.76 | 14.69 | 22.92 | 14.54 | 26.17 | 15.29 |
| 2014 | 6.89 | 10.82 | 7.91 | 10.33 | 9.61 | 11.99 | 20.01 | 13.54 | 22.26 | 14.13 | 26.18 | 15.74 |
| Basic childcare |  |  |  |  |  |  |  |  |  |  |  |  |
| 2002 | 4.61 | 7.43 | 3.31 | 6.26 | 2.76 | 7.55 | 11.74 | 11.61 | 6.47 | 9.34 | 3.22 | 6.52 |
| 2008 | 4.79 | 6.45 | 3.45 | 5.78 | 2.46 | 5.42 | 13.40 | 12.72 | 7.48 | 9.66 | 3.49 | 5.61 |
| 2014 | 5.06 | 8.06 | 5.82 | 8.49 | 3.14 | 6.02 | 10.20 | 11.34 | 9.63 | 11.97 | 3.47 | 6.50 |
| Quality childcare |  |  |  |  |  |  |  |  |  |  |  |  |
| 2002 | 4.67 | 6.54 | 3.08 | 5.24 | 1.79 | 4.49 | 4.26 | 5.85 | 3.13 | 5.65 | 2.15 | 4.60 |
| 2008 | 4.72 | 5.66 | 4.25 | 6.38 | 1.64 | 3.73 | 4.15 | 6.67 | 3.45 | 5.69 | 1.78 | 3.99 |
| 2014 | 5.64 | 8.12 | 5.51 | 7.78 | 2.54 | 5.37 | 5.12 | 7.20 | 4.15 | 5.68 | 2.32 | 4.31 |
| Leisure |  |  |  |  |  |  |  |  |  |  |  |  |
| 2002 | 29.66 | 19.68 | 32.57 | 21.68 | 35.12 | 21.17 | 21.66 | 16.18 | 20.74 | 16.59 | 25.26 | 17.96 |
| 2008 | 25.78 | 19.45 | 31.91 | 21.24 | 37.12 | 24.15 | 21.37 | 16.71 | 23.15 | 17.23 | 24.21 | 17.15 |
| 2014 | 29.79 | 20.34 | 31.97 | 19.89 | 35.50 | 21.63 | 20.94 | 15.60 | 23.19 | 17.28 | 26.59 | 18.60 |
| Obs. | 385 |  | 1512 |  | 992 |  | 695 |  | 1727 |  | 467 |  |

# Table A1.5 – OLS Results – Coefficients of control variables - weekly hours

|  | (1) | (2) | (3) | (4) | (5) |
| --- | --- | --- | --- | --- | --- |
| VARIABLES | Market work | Household work | Basic childcare | Quality childcare | Leisure |
| Age range 35-44 | 0.14 | 0.26 | -0.45 | 0.51* | 0.41 |
|  | (1.330) | (0.501) | (0.379) | (0.262) | (0.769) |
| Age range 45-54 | -3.91** | 2.27*** | -0.41 | -0.22 | 2.20** |
|  | (1.749) | (0.658) | (0.433) | (0.302) | (1.058) |
| University | -5.78*** | -1.49*** | 1.68*** | 0.38* | 3.26*** |
|  | (1.160) | (0.448) | (0.308) | (0.219) | (0.729) |
| South | 3.92*** | -0.85** | -0.48** | -0.81*** | 0.08 |
|  | (1.091) | (0.378) | (0.236) | (0.159) | (0.672) |
| Number of children | -3.86*** | 0.65 | 7.35*** | 2.27*** | -3.86*** |
|  | (1.346) | (0.478) | (0.427) | (0.286) | (0.796) |
| Children 3-5 y.o. | 3.34** | -0.01 | -4.26*** | -1.16*** | -0.09 |
|  | (1.498) | (0.509) | (0.404) | (0.314) | (0.862) |
| Children 6-10 y.o. | 3.08** | 0.54 | -6.02*** | -2.05*** | 2.18*** |
|  | (1.337) | (0.484) | (0.390) | (0.279) | (0.794) |
| Children 11-14 y.o. | 3.85*** | 1.63*** | -7.07*** | -3.42*** | 2.99*** |
|  | (1.334) | (0.479) | (0.391) | (0.264) | (0.804) |
| Industry | 0.75 | -2.64*** | 0.63 | 0.34 | 0.71 |
|  | (2.138) | (0.818) | (0.401) | (0.326) | (1.266) |
| Constructions | -3.92 | -2.59*** | 0.54 | 0.66 | 4.71*** |
|  | (2.672) | (1.000) | (0.546) | (0.440) | (1.665) |
| Services | 0.29 | -2.37*** | 0.82** | 0.66** | 0.73 |
|  | (2.049) | (0.805) | (0.388) | (0.314) | (1.205) |
| Constant | 38.69*** | 7.05*** | -0.48 | 2.11*** | 33.67*** |
|  | (2.700) | (1.002) | (0.590) | (0.459) | (1.581) |
| Obs. | 5,778 | 5,778 | 5,778 | 5,778 | 5,778 |

Sample of full-time working parents selected from ISTAT “Indagine Multiscopo sulle famiglie – Uso del tempo”, years 2002, 2008, 2014. Observations on weekdays and weekend days diaries are pooled together. The dependent variable is the weekly time spent in the different categories, in hours. Results on gender gaps by year are reported in Tables 3-7. Reference categories: Age Range 22-34, No University degree, Children 0-2 y.o., Agriculture. Standard errors are clustered at the family level. *significant a 10%, **significant at 5%, ***significant at 1%.

2
