# Supplementary material for: The Gender Gaps in Time-Use Within Italian Households During 2002–2014
Source: Ital Econ J. 2022 Oct 21:1–34. Online ahead of print. doi: 10.1007/s40797-022-00211-5 (PMC9589588; doi:10.1007/s40797-022-00211-5)
Supplement: Supplementary file 2 — Supplementary Material 2 [file 40797_2022_211_MOESM2_ESM.docx]

Appendix 2

# Table A2.1 – OLS results -Gender gap in Market Work

# Hours per Week

Year Male Average Gender Gap

(Female-Male)

| 2002  2008 | 38.28  38.29 | -38.283^∗∗∗^ (0.920)  -38.291^∗∗∗^ | -47.147^∗∗∗^ (2.494)  -47.134^∗∗∗^ |
| --- | --- | --- | --- |
| 2014 | 34.52 | (1.065)  -34.523^∗∗∗^ | (2.575)  -42.961^∗∗∗^ |
| Change 2014-2002 |  | (1.192) 3.760^∗∗^ (1.506) | (2.598)  4.187^∗∗∗^ (1.508) |
| Controls Obs. | 6456 | NO 6456 | YES 6456 |

Sample of traditional families in which only the father works full time, selected from the ISTAT “Indagine Multiscopo sulle famiglie – Uso del tempo”, years 2002, 2008, 2014. Observations on weekdays and weekend days diaries are pooled together. The dependent variable is the weekly time in hours obtained multiplying the observed daily time by 7. Column (3): gender gaps estimated from equation (1) without controls; column (4): gender gaps estimated controlling for: age class, education level, geographical area of residence, number if children, number of children in different age ranges, sector of employment. Standard errors are clustered at the family level. *significant a 10%, **significant at 5%, ***significant at 1%.

# Table A2.2 – OLS results -Gender gap in Household Work

# Hours per week

Year Male Average Gender Gap

(Female-Male)

| 2002  2008 | 5.30  5.22 | 33.672^∗∗∗^ (0.506)  32.335^∗∗∗^ | 35.532^∗∗∗^ (0.931)  34.197^∗∗∗^ |
| --- | --- | --- | --- |
| 2014 | 5.80 | (0.585)  29.033^∗∗∗^ | (1.030)  30.761^∗∗∗^ |
| Change 2014-2002 |  | (0.630)  -4.639^∗∗∗^ (0.808) | (0.989)  -4.771^∗∗∗^ (0.815) |
| Controls Obs. | 6456 | NO 6456 | YES 6456 |

Sample of traditional families in which only the father works full time, selected from the ISTAT “Indagine Multiscopo sulle famiglie – Uso del tempo”, years 2002, 2008, 2014. Observations on weekdays and weekend days diaries are pooled together. The dependent variable is the weekly time in hours obtained multiplying the observed daily time by 7. Column (3): gender gaps estimated from equation (1) without controls; column (4): gender gaps estimated controlling for: age class, education level, geographical area of residence, number if children, number of children in different age ranges, sector of employment. Standard errors are clustered at the family level. *significant a 10%, **significant at 5%, ***significant at 1%.

# Table A2.3– OLS results- Gender gap in Basic Childcare

# Hours per week

Year Male Average Gender Gap

(Female-Male)

| 2002  2008 | 2.08  2.41 | 7.266^∗∗∗^ (0.282)  8.087^∗∗∗^ | 8.296^∗∗∗^ (0.425)  9.113^∗∗∗^ |
| --- | --- | --- | --- |
| 2014 | 2.94 | (0.344)  7.515^∗∗∗^ | (0.458)  8.623^∗∗∗^ |
|  |  | (0.381) | (0.491) |
| Change 2014-2002 |  | 0.250 | 0.327 |
|  |  | (0.475) | (0.476) |
| Controls Obs. | 6456 | NO 6456 | YES 6456 |

Sample of traditional families in which only the father works full time, selected from the ISTAT “Indagine Multiscopo sulle famiglie – Uso del tempo”, years 2002, 2008, 2014. Observations on weekdays and weekend days diaries are pooled together. The dependent variable is the weekly time in hours obtained multiplying the observed daily time by 7. Column (3): gender gaps estimated from equation (1) without controls; column (4): gender gaps estimated controlling for: age class, education level, geographical area of residence, number if children, number of children in different age ranges, sector of employment. Standard errors are clustered at the family level. *significant a 10%, **significant at 5%, ***significant at 1%.

# Table A2.4 – OLS results Gender gap in Quality Childcare

# Hours per Week

Year Male Average Gender Gap

(Female-Male)

| 2002  2008 | 2.86  2.87 | 1.263^∗∗∗^ (0.195)  1.282^∗∗∗^ | 1.762^∗∗∗^ (0.413)  1.753^∗∗∗^ |
| --- | --- | --- | --- |
| 2014 | 3.27 | (0.225)  1.424^∗∗∗^ | (0.413)  1.843^∗∗∗^ |
|  |  | (0.263) | (0.440) |
| Change 2014-2002 |  | 0.160 | 0.081 |
|  |  | (0.328) | (0.329) |
| Controls Obs. | 6456 | NO 6456 | YES 6456 |

Sample of traditional families in which only the father works full time, selected from the ISTAT “Indagine Multiscopo sulle famiglie – Uso del tempo”, years 2002, 2008, 2014. Observations on weekdays and weekend days diaries are pooled. The dependent variable is the weekly time in hours obtained multiplying the observed daily time by 7. Column (3): gender gaps estimated from equation (1) without controls; column (4): gender gaps estimated controlling for: age class, education level, geographical area of residence, number if children, number of children in different age ranges, sector of employment. Standard errors are clustered at the family level. *significant a 10%, **significant at 5%, ***significant at 1%.

# Table A2.5 – OLS results - Gender gap in Leisure

# Hours per Week

Year Male Average Gender Gap

(Female-Male)

| 2002  2008 | 33.97  34.32 | -7.747^∗∗∗^ (0.542)  -7.462^∗∗∗^ | -6.688^∗∗∗^ (1.587)  -6.413^∗∗∗^ |
| --- | --- | --- | --- |
| 2014 | 34.76 | (0.621)  -6.874^∗∗∗^ | (1.634)  -5.880^∗∗∗^ |
|  |  | (0.719) | (1.653) |
| Change 2014-2002 |  | 0.873 | 0.808 |
|  |  | (0.900) | (0.905) |
| Controls Obs. | 6456 | NO 6456 | YES 6456 |

Sample of traditional families in which only the father works full time, selected from the ISTAT “Indagine Multiscopo sulle famiglie – Uso del tempo”, years 2002, 2008, 2014. Observations on weekdays and weekend days diaries are pooled together. The dependent variable is the weekly time in hours obtained multiplying the observed daily time by 7. Column (3): gender gaps estimated from equation (1) without controls; column (4): gender gaps estimated controlling for: age class, education level, geographical area of residence, number if children, number of children in different age ranges, sector of employment. Standard errors are clustered at the family level. *significant a 10%, **significant at 5%, ***significant at 1%.

# Table A2.6- Weekly OLS Results - Covariates

|  | (1) | (2) | (3) | (4) | (5) |
| --- | --- | --- | --- | --- | --- |
| VARIABLES | Market work | Household work | Basic childcare | Quality childcare | Leisure |
| Age range 35-44 | -1.47** | 2.12*** | -1.36*** | 0.33 | 0.56 |
|  | (0.732) | (0.451) | (0.309) | (0.223) | (0.637) |
| Age range 45-54 | -2.83*** | 3.77*** | -1.11*** | -0.45* | 1.51 |
|  | (1.090) | (0.632) | (0.362) | (0.270) | (0.931) |
| University | 0.48 | -2.71*** | 1.08** | 0.78** | 2.00** |
|  | (1.233) | (0.581) | (0.464) | (0.313) | (0.954) |
| South | 1.71*** | 1.10*** | -0.31 | -0.69*** | 0.06 |
|  | (0.607) | (0.357) | (0.219) | (0.158) | (0.573) |
| Number of children | -0.93 | -1.14*** | 5.84*** | 2.39*** | -3.64*** |
|  | (0.728) | (0.390) | (0.314) | (0.216) | (0.652) |
| Children 3-5 y.o. | 0.65 | 0.82* | -3.08*** | -1.56*** | 1.06 |
|  | (0.840) | (0.465) | (0.350) | (0.251) | (0.762) |
| Children 6-10 y.o. | 0.33 | 1.68*** | -4.72*** | -1.89*** | 2.45*** |
|  | (0.772) | (0.419) | (0.305) | (0.228) | (0.706) |
| Children 11-14 y.o. | 1.92** | 2.92*** | -5.86*** | -2.94*** | 2.05*** |
|  | (0.780) | (0.402) | (0.288) | (0.218) | (0.691) |
| Industry | -10.53*** | 1.98** | 1.04*** | 0.88** | 0.73 |
|  | (2.626) | (0.883) | (0.380) | (0.409) | (1.668) |
| Constructions | -8.88*** | 0.90 | 1.26*** | 0.25 | 1.64 |
|  | (2.780) | (0.925) | (0.394) | (0.408) | (1.787) |
| Services | -7.65*** | 1.03 | 1.53*** | 0.46 | 0.54 |
|  | (2.551) | (0.835) | (0.359) | (0.385) | (1.620) |
| Constant | 47.27*** | 0.58 | -0.98* | 1.75*** | 35.67*** |
|  | (2.739) | (1.017) | (0.536) | (0.492) | (1.865) |
| Obs. | 6,456 | 6,456 | 6,456 | 6,456 | 6,456 |

Sample of traditional families in which only the father works full time, selected from the “Indagine Multiscopo sulle famiglie – Uso del tempo”, years 2002, 2008, 2014. Observations on weekdays and weekend days diaries are pooled together. The dependent variable is the weekly time in hours spent in the different categories. Results on gender gaps by year are reported in Tables A2.1-A2.5. Reference categories: Age Range 22-34, No University degree, Children 0-2 y.o., Agriculture. Standard errors are clustered at the family level. *significant a 10%, **significant at 5%, ***significant at 1%.

# Table A2.7a Descriptive Statistics – Time categories by gender -

# Sample of traditional families

| Market work |  | | | |
| --- | --- | --- | --- | --- |
| 2002 | 38.28 | 33.97 | 0.00 | 0.00 |
| 2008 | 38.29 | 34.37 | 0.00 | 0.00 |
| 2014 | 34.52 | 34.20 | 0.00 | 0.00 |
| Household work |  |  |  |  |
| 2002 | 5.30 | 10.54 | 38.97 | 16.77 |
| 2008 | 5.22 | 10.29 | 37.55 | 16.51 |
| 2014 | 5.80 | 10.39 | 34.83 | 15.86 |
| Basic childcare |  |  |  |  |
| 2002 | 2.08 | 5.28 | 9.35 | 10.61 |
| 2008 | 2.41 | 5.31 | 10.50 | 11.31 |
| 2014 | 2.94 | 6.50 | 10.46 | 11.27 |
| Quality childcare |  |  |  |  |
| 2002 | 2.86 | 5.50 | 4.12 | 6.04 |
| 2008 | 2.87 | 5.43 | 4.15 | 6.27 |
| 2014 | 3.27 | 5.81 | 4.69 | 6.53 |
| Leisure |  |  |  |  |
| 2002 | 33.97 | 21.60 | 26.22 | 16.02 |
| 2008 | 34.32 | 21.92 | 26.85 | 15.87 |
| 2014 | 34.76 | 21.50 | 27.89 | 15.60 |
| Obs. | 6456 |  | 6456 |  |
|  |  |  |  |  |

Men Women Mean sd Mean sd

# Table A2.7b Descriptive Statistics – Covariates - Years 2002, 2008, 2014 pooled - Sample of traditional families

| Variable | Mean | sd |
| --- | --- | --- |
| Woman | 0.50 | 0.50 |
| Age Range 25-34 | 0.24 | 0.43 |
| Age Range 35-44 | 0.55 | 0.50 |
| Age Range 45-54 | 0.21 | 0.41 |
| University | 0.08 | 0.26 |
| South | 0.55 | 0.50 |
| Number of Children | 1.62 | 0.72 |
| Employed | 0.50 | 0.50 |
| Part-time | 0.00 | 0.00 |
| Married | 1.00 | 0.00 |
| Children 0-2 y.o. | 0.28 | 0.50 |
| Children 3-5 y.o. | 0.32 | 0.52 |
| Children 6-10 y.o. | 0.48 | 0.61 |
| Children 11-14 y.o. | 0.53 | 0.63 |
| Weekend | 0.64 | 0.48 |
| Agriculture | 0.03 | 0.17 |
| Industry | 0.15 | 0.36 |
| Construction | 0.09 | 0.29 |
| Services | 0.22 | 0.42 |
| Obs. | 6456 |  |
